# Mapping Regional Distribution of a Single Tree Species: Whitebark Pine in the Greater Yellowstone Ecosystem

**DOI:** 10.3390/s8084983

**Published:** 2008-08-25

**Authors:** Lisa Landenburger, Rick L. Lawrence, Shannon Podruzny, Charles C. Schwartz

**Affiliations:** 1 Land Resources and Environmental Sciences Department, P.O. Box 173490, Montana State University, Bozeman, MT 59717-3490, U.S.A.; E-mail: lisa_landenburger@usgs.gov (L.L.); 2 USGS Northern Rocky Mountain Science Center, Bozeman, MT 59717, U.S.A.; E-mails: shannon_podruzny@usgs.gov (S.P.); chuck_schwartz@usgs.gov (C.C.S.)

**Keywords:** Remote sensing, Landsat, Yellowstone, See5, classification trees, boosting

## Abstract

Moderate resolution satellite imagery traditionally has been thought to be inadequate for mapping vegetation at the species level. This has made comprehensive mapping of regional distributions of sensitive species, such as whitebark pine, either impractical or extremely time consuming. We sought to determine whether using a combination of moderate resolution satellite imagery (Landsat Enhanced Thematic Mapper Plus), extensive stand data collected by land management agencies for other purposes, and modern statistical classification techniques (boosted classification trees) could result in successful mapping of whitebark pine. Overall classification accuracies exceeded 90%, with similar individual class accuracies. Accuracies on a localized basis varied based on elevation. Accuracies also varied among administrative units, although we were not able to determine whether these differences related to inherent spatial variations or differences in the quality of available reference data.

## 1. Introduction

Whitebark pine (*Pinus albicaulis* Engelm., WBP) seeds have long been identified as an important food source for grizzly bears (*Ursus arctos*) in the Greater Yellowstone Ecosystem (GYE) and are, therefore, an important element of suitable grizzly bear habitat [1]. WBP also serves as a keystone species because its presence increases the biodiversity of both plant and animal communities throughout the ecosystem [2]. The overall health and status of WBP is currently threatened by infestation by mountain pine beetle (*Dendroctonus ponderosae*) and the spread of whitepine blister rust (*Cronartium ribicola*).

Mapping WBP distribution is integral to the success of long-term monitoring since, before we can study, understand and mitigate the mechanisms driving destructive agents of WBP, we must first know its distribution across the landscape. Several factors, however, make mapping WBP within the GYE difficult. This area encompasses approximately 57,000 km^2^, making consistent ground mapping within a reasonable time infeasible. Ground mapping efforts have been conducted over several decades by the national forests and national parks administering the area, but methods and efforts have not been consistent, and the time required has made this approach impractical for monitoring current threats. Satellite remote sensing has the potential to provide synoptic coverage of the area. Even for moderate resolution imagery, such as Landsat, several images are required to cover this area. Such imagery, however, historically has been deemed inappropriate for conducting species-level mapping [3]. Previous efforts to map WBP in the northern Rockies met with low accuracies [4, 5]. We believed that these low accuracies might be a result of several factors, including (1) lack of adequate training data to represent the wide variability of this species across the region, (2) mapping WBP concurrently with other land cover types, resulting in approaches that might have compromised accuracy of the WBP class to increase overall accuracy and relative accuracy across all classes, and (3) use of traditional classification algorithms that are less accurate than some more recent algorithms.

The Interagency Grizzly Bear Study Team initiated an effort to map the distribution of WBP throughout the GYE in the fall of 2003. We sought to determine whether an approach focusing on a single species and using recent advances in classification methods could result in increased accuracies over those previously reported.

## 2. Methods

**Figure 1.**
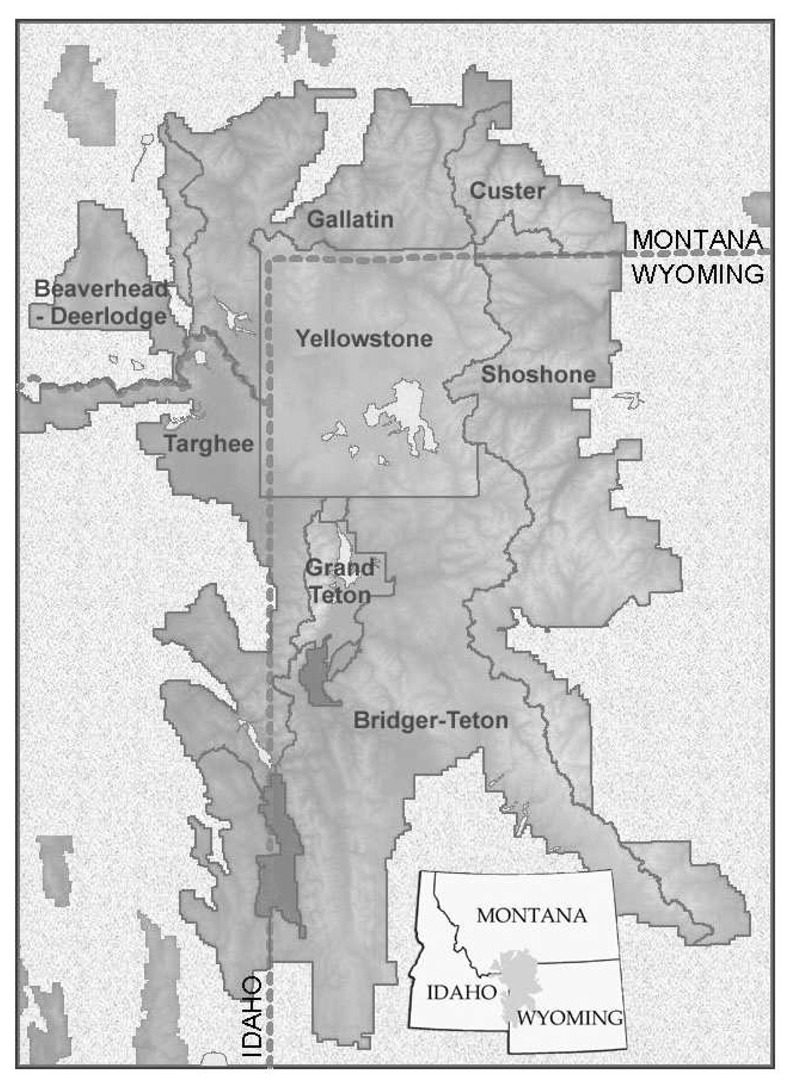
Location of study area, showing administrative units within the national forest and national park systems.

**Figure 2.**
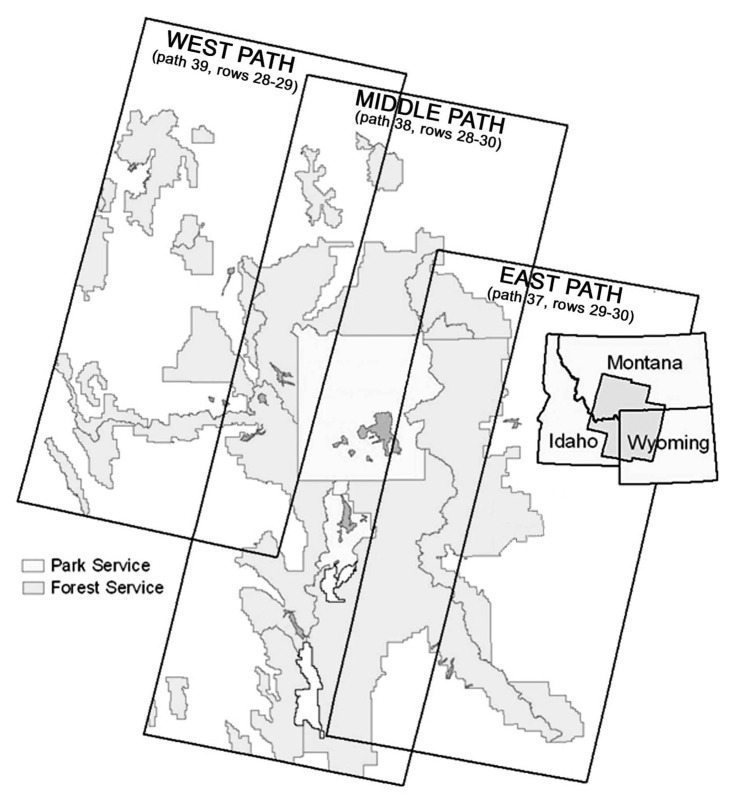
Study area classification divisions based on east, west and middle paths of Landsat ETM+ satellite imagery, including national forest and national park boundaries.

Our study area covered the GYE, including portions of six national forests and all of two national parks (Figure 1). Landsat 7 Enhanced Thematic Mapper Plus (ETM+) satellite imagery was used as the primary mapping data source. Seven ETM+ scenes for September 1999 covering the core of the GYE (Figure 2) were provided with geometric and radiometric corrections by the EROS Data Center, Sioux Falls, South Dakota.

We intended for reference data to use information collected by U.S. Forest Service and National Park Service in conjunction with their standard timber-stand exams, vegetation plots, soil surveys, and other field activities, because the extent of the study area made extensive ground collection impractical. The agencies responded well to our requests for data, and we were able to compile a large pool of vegetation data that collectively constituted a fairly sufficient representation of the spatial complexities of the ecosystem. The types and amount of information recorded for these data varied greatly due to multiple data sources and differing purposes for which the data were collected. For those locations where the percent of WBP present in the canopy was recorded, we considered WBP present for the purposes of our study if whitebark pine accounted for 25% or more of the upper canopy and thus was visible from a satellite.

The reference data also exhibited varying degrees of spatial accuracy. Some data were collected without GPS units, and various methods were used in estimating ground locations resulting in disparate degrees of spatial accuracy. A substantial portion of the data collected with GPS also had considerable error (up to 300 m), due to selective availability and the lack of post-differential correction. Locational accuracy commensurate with the Landsat 30-m resolution was required. Spatial reliability checks were performed on each of the over 7,000 collected locations by overlaying datum on digital orthographic quads (DOQs). Aerial photos were used to correct locations over distances ranging from ten to a few hundred meters to their most probable location based on characteristics recorded with the data and identification of WBP stands on the photos. Points that could not be corrected with a high degree of certainty (29% of the collected data) were eliminated from the analysis. We were also able to generate additional reference data using aerial photos (8,000+ points) accessed from Forest Service offices across the GYE. A total of 15,110 training data points, excluding random points generated in the image overlap areas, were compiled. Photo-interpreted points comprised 54% and agency supplied field data comprised 46%. 85% of our reference data locations were selected randomly for classification model development, while the remaining 15% was reserved for accuracy assessment.

Spectral and spectrally derived predictor variables used in our classifications included (1) at-satellite reflectances scaled to 8-bit values by EROS Data Center for the six ETM+ reflective bands (the thermal band was not provided), (2) re-scaled at-satellite tasseled cap brightness, greenness, and wetness values [6], (3) principal component data values for all six bands, and (4) normalized difference vegetation index (NDVI), where NDVI = (near infrared – red)/(near infrared + red). The derived predictor variables do not provide additional information beyond what is contained in the original spectral bands, but we included them because they have been shown to be well correlated to vegetation types and might be used by the classification algorithm to map types more efficiently than the original bands. Ancillary data considered to have strong predictive powers for WBP occurrence and included as predictor variables included latitude and three data layers derived from the USGS 30-m National Elevation Dataset Digital Elevation Models, including elevation, slope, and aspect. Aspect was transformed by taking the cosine of aspect in radians and stretching it to 0 - 200 by adding 1 and multiplying by 200. Latitude was generated from a 1-km regular grid and then re-sampled to 30 m (Δ latitude ≅ 0.00011 degrees per km).

Classification tree analysis (CTA) has been shown to be an effective tool for classification of remotely sensed data in conjunction with ancillary data [7]. CTA examines the input reference observations (populated with predictor variable values) and recursively partitions the data based on binary splits of individual predictor variables such that deviance in the response variable is minimized [8]. We used the See5 software program for our analysis [9, 10]. A potential advantage to See5 was the option for boosting, a technique reported to significantly reduce the training error and enhance the classification accuracy [11, 12]. Boosting generated a user-specified number of classification trees such that each successive tree attempted to correct misclassification of the previous tree [13]. The final predicted classification was based on a plurality vote from the complete set of classification trees. We used the maximum of 99 boosts provided by the program based on previous statistical research [12]. The development by the USGS of an interface between See5 and ERDAS Imagine made it particularly useful when compared to other boosting algorithms.

Classifications were conducted separately on three sets of images covering the study area (Figure 2), the middle-path (path 38, rows 28-30), the east-path (path 37, rows 29-30), and the west-path (path 39, rows 28-29). Classification was first performed on the middle path, yielding high accuracy rates that justified using the classification results of the middle-path in areas of path-overlap to identify supplemental training samples for the classification of the east- and west-paths [14]. This method ensured a smooth and seamless transition across the final merged classified image. Four thousand random points in each overlap area were generated and populated with the corresponding classification codes from the middle-path results. These points were added to the training samples for the east- and west-paths, respectively. Accuracy was assessed using the reserved 15% of the reference data.

**Figure 3.**
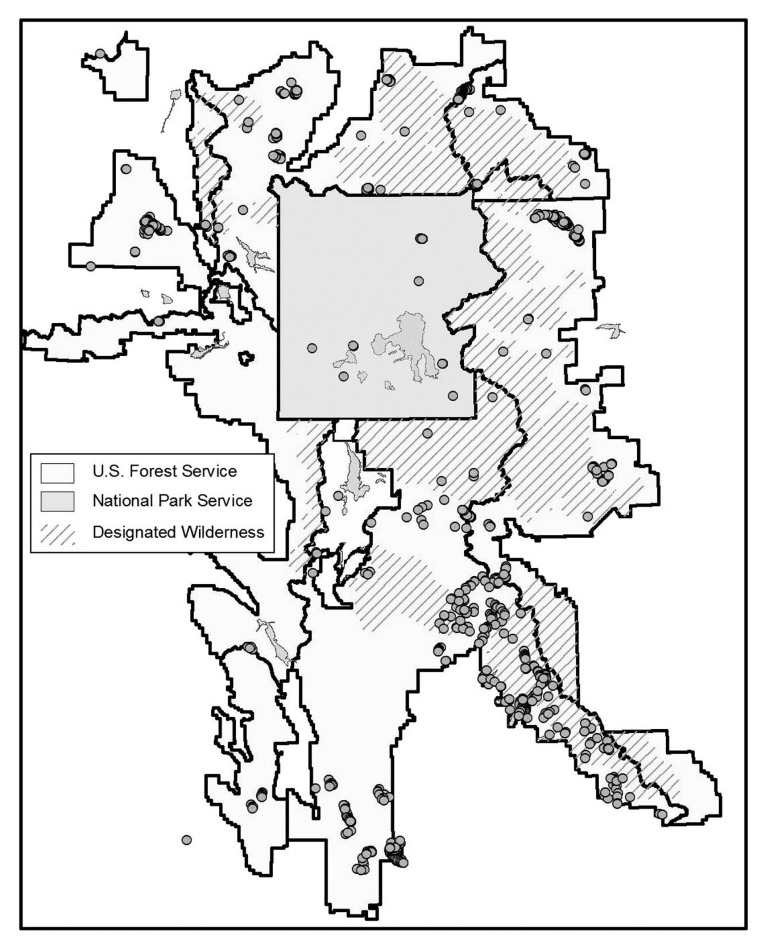
Distribution of field validation points across the Greater Yellowstone Ecosystem.

We conducted an additional field-based accuracy assessment to analyze the sensitivity of the analysis to varying densities of WBP, the effects of elevational gradients on map accuracy, and the variation of accuracy associated with different data sources. Sampling strategy was dictated by the size of the study area (approximately 57,000 km^2^), fiscal limitations, time constraints, and inaccessibility of sizeable roadless wilderness areas within the ecosystem. A subset for field investigation was selected from the total number of sites predicted as WBP. These field sites were stratified using distance from nearest road (≤ 4 miles) as well as wide geographic coverage. Data were also collected on a stand scale by mapping timber stands of close proximity to field sites on aerial photographs. GPS data collected by the Bridger Teton National Forest, the GAP project, and the Inter-Agency Whitebark Pine Monitoring Program were also included to augment the field data collected. The resulting field validation points were distributed throughout the study area, but tended to cluster, making them potentially inadequate for testing overall map accuracy (Figure 3). They were used, rather, for evaluating variations in accuracy related to WBP density, elevation, and geographic location.

## 3. Results

**Figure 4.**
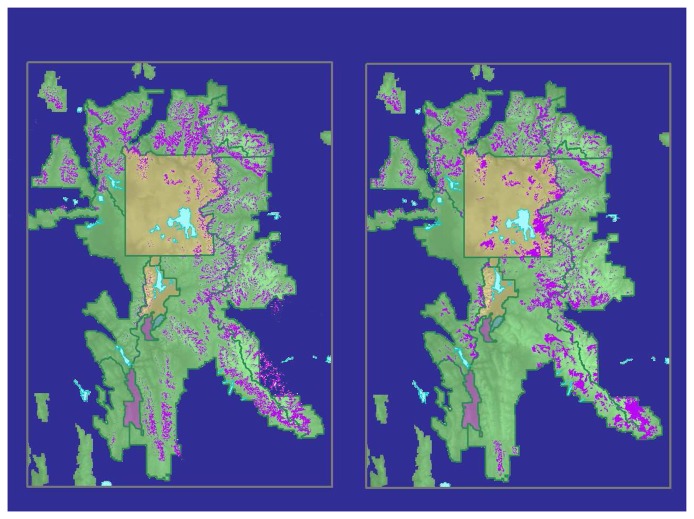
(a) Final classified image of whitebark pine distribution within the GYE (left) and (b) compilation of previously mapped WBP locations within the Greater Yellowstone Ecosystem (right). Whitebark pine is mapped in magenta.

Classification of the middle path resulted in overall accuracy of 95.8%, with similarly high class accuracies (Table 1). Classification of the east and west paths provided substantially similar accuracies, except that producer's accuracy for the west path resulted in more errors of omission for WBP and fewer errors of omission for non-WBP (Table 1). The final classified image (Figure 4a) yielded an overall accuracy of 95.7% and a user's class accuracy for WBP of 92.9%. The KHAT statistic was calculated at 0.90.

The classification showed substantial differences from a map that combined existing USFS and NPS maps of WBP distribution (Figure 4b). The existing maps used a variety of techniques and minimum mapping units, so a direct comparison was not appropriate. Close visual inspection of the resulting map revealed non-forested areas in the higher elevations (> 2,900 m) that were misclassified as WBP.

A series of accuracy assessments using field validation data was conducted to determine a threshold value of percent WBP needed in the upper canopy to delineate “presence” versus “absence” (Table 2). Each assessment assumed a different threshold value for WBP. The defining threshold was determined as the value that returned the highest accuracy of the predictive model. A threshold value of 15% optimized the user's accuracy for both WBP and non-WBP (83.1% and 83.3%, respectively) while maintaining a 91.4% producer's accuracy and 83.2% for overall classification accuracy. Locations with less than 10% to 15% WBP in the canopy were often classified as non-WBP at low thresholds, while areas with less than 15% to 20% were generally correctly classified as WBP, resulting in lower accuracies at higher thresholds.

A natural breaks classification based on the Jenk's optimization was applied to partition the validation points into six discrete elevation classes of minimal variance [15]. Accuracy assessments of field validation data were conducted at each of these elevation ranges (Table 3). Inspection of the error matrices associated with the mid-to-high elevations (ranges 3 – 6) consistently demonstrated lower accuracies for non-WBP than WBP. WBP was over-predicted at these elevations. At lower elevations (ranges 1 and 2), however, where WBP tends to represent a relatively low presence in mixed coniferous stands, the classification tended to under predict WBP presence.

We also conducted separate accuracy assessments based on the field validation data for each of the national parks and national forests included in the study area (Table 4). Caribou-Targhee National Forest and the national parks are included for completeness, but are of limited value as no WBP present stands were sampled in those jurisdictions. Substantial differences in accuracy existed. The Shoshone National Forest on the eastern side of the study area (Figure 1) had the lowest overall accuracy (74.5%), while the Beaverhead National Forest on the northwest corner of the study area (Figure 1) had the highest overall accuracy (94.4%). All other accuracies were in the 83% to 88% range.

## 4. Discussion

Our classifications resulted in very high accuracy rates, demonstrating considerable success in detecting WBP in pure and mixed stands. This was especially notable considering Landsat is generally not expected to be adequate for classification at the species level, and previous attempts had not been successful. There are several factors that we believe were important in our classification success, although it is not possible to quantify the impact of the factors individually.

We used classification algorithms that have been recently developed and applied to remotely sensed data [16]. Classification tree analysis generally has resulted in improved accuracies when compared to other classification accuracy, and boosting algorithms have been commonly reported to increase classification accuracies by 10% or more compared to non-boosted classification trees, although increased accuracy is not guaranteed [13]. We used a simple boosting algorithm [10]; it is possible that recent advances in boosting and related bagging algorithms might have further improved accuracies [13, 16]. A disadvantage of using boosting was that it resulted in no single classification tree that could be interpreted to evaluate how the algorithm successfully distinguished WBP from other conifer species.

We also believe that focusing on a single class might have improved our accuracies compared to previous classifications. Classification of multiple land cover types in a single classification necessarily entails trade-offs; an approach that improves accuracy of one class might decrease accuracy of another class. We were able to select from among multiple algorithms and approaches and select the one that would improve WBP accuracy without concern for other species. Results of other classification methods are not included in this paper because this was not a rigorous study of algorithms, but multiple approaches were examined.

We also were able to assemble an extensive reference data set as a result of a high level of cooperation from national forests and national parks within our study area, as well as the availability of excellent aerial photography coverage. These data required extensive review and filtering to make them acceptable for use in a remote sensing study, and again high levels of cooperation for local land managers was extremely valuable in this process.

A fourth factor that might have been important in our success compared to other species-level studies with Landsat data was the spatial distribution of WBP. Exploratory data analysis of our results indicated that elevation was the most important predictor variable for the occurrence of WBP. WBP distribution is heavily controlled by elevation and it can occupy nearly pure homogeneous stands in harsh, dry, windy mountainous terrain, although it typically co-exists with other conifers in moister and more protected high-elevation sites [17]. This elevation control on distribution likely reduced species confusion with other pines, which typically exist at lower elevations within the study area.

Elevation also created issues for our classification. WBP can be completely out-competed by subalpine fir and Engleman spruce in localized areas of higher moisture, for example along drainages. Our model over predicted WBP by 20% in these high elevation sites (Table 3). Adding a hydrologic index as a predictor variable might improve accuracy for these sites. Bedrock geology, geomorphology, and soil types also impact WBP distribution [18] and might be evaluated for future classifications.

We also noted differences in accuracy across our study area associated with different national forests and national parks. The reasons for these spatial differences in accuracy were unclear, but we believe there were multiple possibilities. These variations might have been a function of differences in the quality of reference data from each jurisdiction. Lower quality data might have less accurately represented the spatial and spectral variability in that location and resulted in a model that did a poorer job in predicting WBP locally. Another possibility was that these differences represented a broader spatial trend in accuracy across our study area. Accuracies tended to be highest on the west side of the study area and lowest on the east side when evaluated by national forest/national park (Table 4). This same trend, however, was not present when accuracy was evaluated by Landsat path (Table 1).

Our comparison of our final map with our compilation of existing maps showed some important differences in WBP distribution (Figure 4). Our map showed greater WBP presence in the southern part of the GYE and generally less in the southern part of Yellowstone National Park. These two maps, however, were derived in very different ways. The compilation of existing maps, in particular, should be viewed with care as it entailed numerous mapping methods and minimum mapping units. Our Landsat-based map was the first to provide consistent coverage at high accuracies across the GYE.

The results of this study are potentially valuable for several on-going efforts, including: (1) GYE Interagency Whitebark Pine Monitory Program from which probabilistic samples will be derived from the WBP map resulting from this study; (2) expansion of efforts to conduct a habitat-based grizzly bear Population Viability Analysis [19, 20], which is currently restricted to areas inside the recovery zone; (3) updates to data layers for the Yellowstone Grizzly Bear Cumulative Effects Model [21, 22]; (4) modeling the potential effects of declines in major food sources or global climate change; (5) use in habitat selection models evaluating the effects of motorized recreation on denning and active grizzly bears; and (6) use in two studies examining GYE carnivore population dynamics that are sponsored by the USGS, National Park Service, and the Wildlife Conservation Society. Other efforts that might benefit include: (1) monitoring the distribution of white pine blister rust in the GYE as part of key foods monitoring required by the grizzly bear recovery plan [20] and conservation strategy [23]; (2) use by state wildlife and federal land agencies for planning and evaluation of management efforts; and (3) distribution through National Biological Information Infrastructure (http://www.nbii.gov/), making this data layer available to the public.

## Figures and Tables

**Table 1.** Comparative accuracies for classification of each path Landsat ETM+ imagery.

**Image Path**	**Producer's**		**User's**		**Overall**
	**WBP**	**Non-WBP**	**WBP**	**Non-WBP**	
middle	93.6%	97.0%	94.1%	96.6%	95.8%
east	94.6%	95.9%	92.7%	97.0%	95.4%
west	89.0%	97.8%	93.7%	96.0%	95.4%

**Table 2.** Accuracy assessments at different presence / absence thresholds for WBP.

**Threshold**	**WBP Producer's Accuracy**	**Non-WBP Producer's Accuracy**	**WBP User's Accuracy**	**Non-WBP User's Accuracy**	**Overall Accuracy**
≥ 5%	84.6%	76.7%	90.9%	64.5%	82.5%
≥ 10%	89.3%	71.8%	85.7%	78.0%	83.2%
≥ 15%	91.4%	69.8%	83.1%	83.3%	83.2%
≥ 20%	91.6%	67.1%	80.8%	84.0%	81.8%
≥ 25%	91.9%	64.7%	78.2%	85.3%	80.5%

**Table 3.** Accuracy assessments at successive elevation ranges.

**Elevation Range**	**WBP Producer's Accuracy**	**Non-WBP Producer's Accuracy**	**WBP User's Accuracy**	**Non-WBP User's Accuracy**	**Overall Accuracy**
Range 1 (7470-8376 m)	0%	100%	NA	95.9%	95.9%
Range 2 (2553-2691 m)	87.0%	83.0%	81.9%	87.8%	84.9%
Range 3 (2692-2805 m)	92.8%	76.0%	91.4%	79.3%	88.3%
Range 4 (2806-2900 m)	92.1%	47.8%	78.4%	74.8%	77.6%
Range 5 (2901-3025 m)	94.4%	43.7%	80.4%	76.0%	79.7%
Range 6 (3026-3104 m)	85.9%	76.2%	89.5%	69.6%	83.0%

**Table 4.** Accuracy assessments for different administrative units.

**National Park or Forest**	**WBP Producer's Accuracy**	**Non-WBP Producer's Accuracy**	**WBP User's Accuracy**	**Non-WBP User's Accuracy**	**Overall Accuracy**
Beaverhead	95.0%	92.7%	97.0%	88.4%	94.4%
Bridger-Teton	86.1%	81.0%	80.1%	86.8%	83.4%
Caribou-Targhee	NA	100.0%	NA	100.0%	100.0%
Custer	87.0%	50.0%	95.5%	24.0%	84.2%
Gallatin	95.6%	61.8%	89.1%	81.0%	87.6%
Shoshone	92.5%	44.7%	73.4%	78.4%	74.5%
Yellowstone & Grand Teton	0.0%	92.3%	0.0%	94.7%	87.8%
